# Abnormal wiring of the connectome in adults with high-functioning autism spectrum disorder

**DOI:** 10.1186/s13229-015-0058-4

**Published:** 2015-12-15

**Authors:** Ulrika Roine, Timo Roine, Juha Salmi, Taina Nieminen-von Wendt, Pekka Tani, Sami Leppämäki, Pertti Rintahaka, Karen Caeyenberghs, Alexander Leemans, Mikko Sams

**Affiliations:** Brain and Mind Laboratory, Department of Neuroscience and Biomedical Engineering, Aalto University, Rakentajanaukio 2 C, FI-02150 Espoo, Finland; iMinds-Vision Lab, Department of Physics, University of Antwerp, Universiteitsplein 1, B-2610 Wilrijk (Antwerp), Belgium; Faculty of Arts, Psychology and Theology, Åbo Akademi University, Fabriksgatan 2, FI-20500 Turku, Finland; Neuropsychiatric Rehabilitation and Medical Centre Neuromental, Kaupintie 11 A, FI-00440 Helsinki, Finland; Clinic for Neuropsychiatry, Department of Psychiatry, Helsinki University Central Hospital, Tukholmankatu 8 F, FI-00290 Helsinki, Finland; Finnish Institute of Occupational Health, Topeliuksenkatu 41, FI-00290 Helsinki, Finland; School of Psychology, Australian Catholic University, Locked Bag 4115, Fitzroy MDC, VIC 3065 Melbourne, Australia; Image Sciences Institute, University Medical Center Utrecht, Heidelberglaan 100, 3584 CX Utrecht, The Netherlands; Advanced Magnetic Imaging Centre, Aalto University, Otakaari 5, FI-02150 Espoo, Finland

**Keywords:** Autism spectrum disorder, Diffusion magnetic resonance imaging, White matter tract, Tractography, Connectivity, Connectome, Brain networks, Graph theoretical analysis

## Abstract

**Background:**

Recent brain imaging findings suggest that there are widely distributed abnormalities affecting the brain connectivity in individuals with autism spectrum disorder (ASD). Using graph theoretical analysis, it is possible to investigate both global and local properties of brain’s wiring diagram, i.e., the connectome.

**Methods:**

We acquired diffusion-weighted magnetic resonance imaging data from 14 adult males with high-functioning ASD and 19 age-, gender-, and IQ-matched controls. As with diffusion tensor imaging-based tractography, it is not possible to detect complex (e.g., crossing) fiber configurations, present in 60–90 % of white matter voxels; we performed constrained spherical deconvolution-based whole brain tractography. Unweighted and weighted structural brain networks were then reconstructed from these tractography data and analyzed with graph theoretical measures.

**Results:**

In subjects with ASD, global efficiency was significantly decreased both in the unweighted and the weighted networks, normalized characteristic path length was significantly increased in the unweighted networks, and strength was significantly decreased in the weighted networks. In the local analyses, betweenness centrality of the right caudate was significantly increased in the weighted networks, and the strength of the right superior temporal pole was significantly decreased in the unweighted networks in subjects with ASD.

**Conclusions:**

Our findings provide new insights into understanding ASD by showing that the integration of structural brain networks is decreased and that there are abnormalities in the connectivity of the right caudate and right superior temporal pole in subjects with ASD.

**Electronic supplementary material:**

The online version of this article (doi:10.1186/s13229-015-0058-4) contains supplementary material, which is available to authorized users.

## Background

Autism spectrum disorder (ASD) is a neurodevelopmental disorder characterized by severe impairments in social interaction and restricted, repetitive patterns of behavior, interests, or activities [1]. It affects almost one percent of the population [2], and the heritability has been estimated to be as high as 90 % [3].

Recent brain imaging findings suggest that there are widely distributed abnormalities affecting the brain connectivity in individuals with ASD [1, 4–6]. First, functional magnetic resonance imaging (MRI) findings indicated reduced long-distance and increased short-distance connectivity in ASD [1, 4, 5]. More recently, diffusion-weighted (DW) MRI has enabled the investigation of in vivo structural brain connectivity, which has led to a significant amount of evidence concerning abnormalities in structural connectivity, reviewed in [6]. However, only a few studies take advantage of the graph theoretical approach to analyze the connectome reconstructed with whole brain tractography in ASD [7–11]. Using graph theoretical analysis, it is possible to investigate both global and local properties of the brain connectivity networks [12, 13]. First, DW MRI data are used to perform whole brain tractography for all subjects. The brain is then divided into small areas called nodes in the network. The white matter tracts connecting the nodes, extracted from the whole brain tractography, are called links or edges. The network can be a binary network, where all of the links have the same weight (one). By contrast, in weighted networks, the links have different weights, defined, for example, based on the number of tracts between the nodes. The network measures used in this study are presented in Table 1.

**Table 1:** The network measures used in this study

Measure	Description
Degree and strength	Degree is the number of links of a node and, thus, also the number of neighbors of the node. Strength is a similar measure for weighted networks: the sum of the weights of the links of a node [12, 13].
Clustering coefficient	Clustering coefficient measures how many of the node’s neighbors are also connected to each other. It is calculated by summing the number of links between the nearest neighbors of the node divided by the maximum possible amount of links between the nearest neighbors [40]. In the weighted networks, the weights of the links are also taken into account [41, 42].
Characteristic path length	Shortest path length is the minimum number of links that are passed through to get from one node to another node. Characteristic path length is the average of the shortest path lengths between each pair of nodes in the network [40]. In the weighted networks, the weights of the links are also taken into account [13].
Efficiency	Global efficiency is the average of the inverse shortest path lengths and is primarily influenced by short paths, whereas the characteristic path length is primarily influenced by long paths. Local efficiency is the efficiency of a subgraph formed by the neighborhood of the node [13, 41, 43, 45].
Betweenness centrality	Betweenness centrality measures the centrality of the node in the network by calculating how many of the network’s shortest paths go through that particular node [46–48].
Hubs	Hubs are the nodes with a big strength or a high betweenness centrality (here defined to be higher than mean + two standard deviations).

The results of the previous DW MRI studies in ASD are inconsistent, as both decreased and increased fiber coherence (i.e., directional consistency within a voxel) measured by fractional anisotropy have been reported [6]. This might be due to differences in the age and cognitive profiles of the subjects, image acquisition parameters, and/or analysis techniques [14–18]. Network analysis has been applied in very few studies so far, most of them investigating children with ASD. First, decreased global clustering coefficient and efficiency were reported in children with ASD [10], indicating decreased segregation and integration of the structural brain networks in ASD. They also studied network properties of Wernicke’s and Broca’s areas and found decreased clustering coefficient in both areas and highly decreased betweenness centrality in Wernicke’s area. This suggests that Wernicke’s area, responsible for comprehension of speech, is less important for the structural brain networks in ASD, which corresponds to known communication deficits in ASD. By contrast, increased clustering coefficient and decreased characteristic path length were reported in children (of approximately the same age) with ASD [11], indicating increased segregation and increased integration. In young adolescents with ASD, no differences in the network measures between patients and controls were found in the structural networks [7]. In only one previous study, adults with ASD were investigated [8]. Density-weighted networks were investigated, and an inverse relation was found between intracranial volume and efficiency in widespread areas in the cortex in both controls and subjects with ASD [8]. In addition, decreased nodal local efficiency was found in ASD in many areas. The same researchers have also investigated brain networks of infants with ASD and reported both globally and locally decreased efficiencies in infants classified with ASD compared to those not classified as ASD [9]. Decreased integration of the structural brain networks in ASD has been found in most of the previous studies, while the other results have significantly differed from each other.

In this study, we performed whole brain tractography for subjects with ASD and age-, gender-, and IQ-matched controls, followed by a complex network analysis [12]. In the previous network studies in ASD, a diffusion tensor model was fitted to the DW MRI data, and then, diffusion tensor imaging (DTI)-based tractography was performed [19, 20]. However, DTI is unable to correctly characterize crossing fiber configurations, which are present in up to 90 % of the white matter tissue [21]. Therefore, high-angular resolution diffusion imaging techniques such as constrained spherical deconvolution (CSD) have been developed. In CSD, multiple fibers passing through a voxel with distinct orientations can be reliably identified [22–25]. For instance, DTI-based and CSD-based tractographies were recently compared in a clinical sample [24]. Corticospinal tracts were investigated in both healthy subjects and patients undergoing presurgical imaging assessment, and it was shown that DTI-based tractography resulted in unreliable and clinically misleading information, whereas CSD-based tractography demonstrated fibers more accurately and improved estimates of safety margins [24]. So far, CSD-based tractography has only been used in three studies in ASD [26–28], but no network analyses were applied in those studies.

Here, we combined CSD-based tractography with graph theoretical analysis to investigate the organization of the structural brain networks in ASD. Based on the previous results in structural brain networks, we expected to find decreased network integration in ASD and hypothesized that the efficiency and normalized characteristic path length of the networks would be decreased in ASD. To investigate the hypothesis of local overconnectivity motivated by previous functional MRI studies, we hypothesized that the segregation of the networks would be increased in ASD and more specifically that the normalized clustering coefficient would be increased in ASD. In addition, we analyzed local properties of the networks to find out whether certain regions would be more important for the information flow in the networks than others.

## Methods

### Participants

The study sample consisted of 14 individuals with ASD and 19 control subjects without any neuropsychiatric disorders. As ASD represents a very heterogeneous group regarding both the genetic background and the spectrum and degree of severity of symptoms, we selected only individuals with Asperger syndrome into our study. Individuals with Asperger syndrome and autism share the same core symptoms, but according to DSM-IV, subjects with Asperger syndrome do not have a clinically significant delay in speech and cognitive development. In DSM-V, this similarity was recognized and autism and Asperger syndrome (among others) were placed on the same spectrum of autistic disorders. Thus, although no qualitative differences should exist, the heterogeneity of ASD implies also etiological heterogeneity.

All subjects were males, and the individuals with ASD were age- and IQ-matched with the controls. To minimize the effect of age-related changes on the neural structure, only individuals aged 40 years or less were eligible for the study [29, 30]. The mean age of individuals with ASD was 28.6 ± 5.7 years and that of controls 26.4 ± 4.7 years. The mean IQs for the ASD and control groups were 125 ± 15 and 128 ± 10, respectively [31]. The patients were recruited from a private neuropsychiatric clinic (NeuroMental) in Helsinki and from the neuropsychiatric clinic in Helsinki University Central Hospital. Only individuals fulfilling ICD-10 (International Classification of Disease; World Health Organization, 1993) criteria, diagnosed by experienced clinicians specialized in developmental neuropsychiatry, were included in the study. Both individuals with ASD and control subjects had a full psychiatric evaluation before inclusion in the study. Diagnostic process for the ASD group included full developmental history, acquired using multiple sources of information (e.g., all previous medical records, parental interviews when possible). Benton Facial Recognition Test [32] and Reading the Mind in the Eyes Test [33] were carried out for all subjects. In addition, all subjects completed autism spectrum quotient [34], empathy quotient [35], and systemizing quotient [36] questionnaires, which had been translated into Finnish, and the translation was confirmed by a back translation [37]. There were no significant differences in total IQ, verbal IQ, performance IQ, Benton Facial Recognition test, Reading the Mind in the Eyes test, or systemizing quotient, whereas individuals with ASD had significantly higher autism spectrum quotient scores (*p* = 0.0000001) and significantly lower empathy quotient scores than controls (*p* = 0.0017) (see Table 1 in [37]). Control subjects were paid for their attendance in the study, and for individuals with ASD, the expenses and the loss of income were compensated. The ethics committee of Hospital District of Helsinki and Uusimaa approved of the research protocol, and all participants signed a written informed consent form before participating in the study.

### Image acquisition

The MR images were acquired with a Signa VH/i 3.0T scanner with HDxt upgrade (General Electric, Milwaukee, WI). A quadrature receiving an eight-channel high-resolution brain array coil was used (MRI Devices Corporation, FL). The maximum field gradient amplitude of the MRI system was 40 mT/m with a slew rate of 150 T/m/s. A high-order shimming with a 24-cm field of view was applied prior to DW imaging. A spin echo pulsed sequence of 60 unique gradient orientations arranged on the unit sphere was used. Eight non-DW B0 images were acquired, and all of the 60 orientations were imaged twice resulting in 120 DW images in total. The *b* value, which controls the diffusion weighting, was 1000 s/mm^2^. Echo time was set to the minimum (approximately 98 ms). Repetition time was 10 s, and the number of excitations was one. The imaging area covered the whole brain with 53 contiguous axial slices. The acquired in-plane resolution of the slices was 1.875 mm × 1.875 mm, and the thickness of the slices was 3.0 mm. The matrix size was 128 × 128. The DW acquisition time was approximately 20 min.

T1-weighted anatomical 3D images were acquired with inversion recovery prepared in a spoiled gradient echo sequence with a resolution of 1 mm × 1 mm × 1 mm using echo time of 2.988 ms; repetition time of 10.02 ms; and flip angle of 15°. Field of view was 256 mm and the matrix size of the volume was 256 × 256 with 178 axial slices.

### Construction of the brain networks

First, the DW images were corrected for subject motion and eddy current-induced distortions [38]. Then, the fiber orientation distribution functions were estimated with CSD, and whole brain tractography was performed in native space for all subjects [23, 39, 40]. Spherical harmonics up to the 6th order were used in the estimation. A fiber orientation distribution threshold of 0.1, a maximum angle deviation of 30 degrees, and a step size of 1 mm were used. The minimum length of the fiber was set to 50 mm. Preprocessing, CSD, and visualization of the networks were performed in *ExploreDTI* [41].

**Fig. 1:**
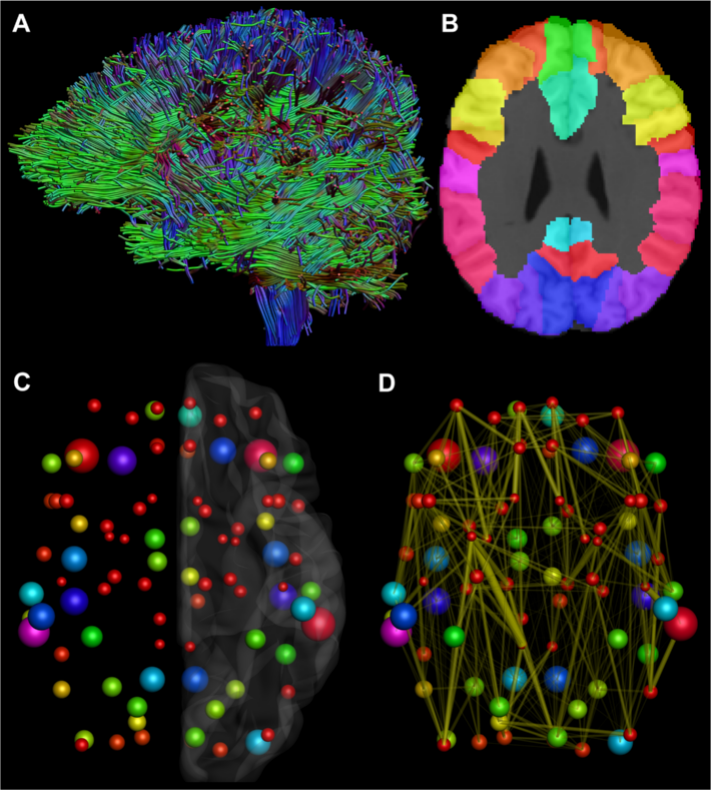
The reconstruction of the structural brain networks. **a** First, a whole-brain constrained spherical deconvolution-based tractography was performed for all subjects. **b** Then, Automated Anatomical Labeling atlas was used to parcellate the brain into 90 regions. **c** These regions become the nodes in the brain networks. The size and color of the nodes correspond to the volume of the region in the Automated Anatomical Labeling atlas. **d** Finally, a link was formed in the brain network, if there was at least one tract between two nodes. The thickness of the links corresponds to the density-weight of the connection, i.e., the number of fibers divided by the mean volume of the two nodes. All of the above steps were performed in the individual space of each subject

**Fig. 2:**
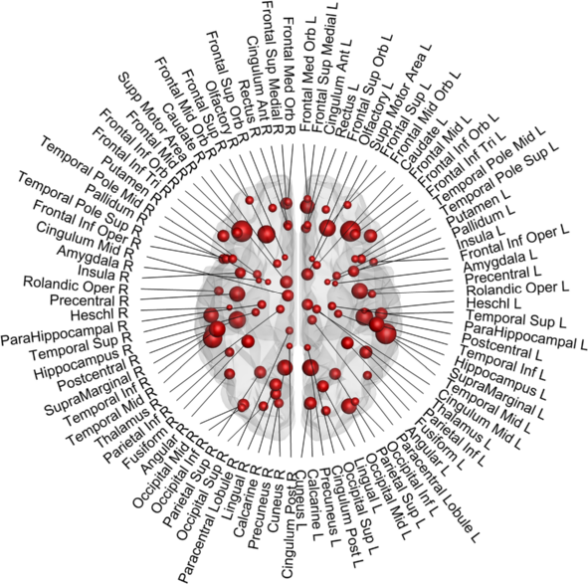
The parcellation of the brain. Automated Anatomical Labeling atlas was used to parcellate the brain into 90 regions. The size of the node corresponds to the volume of the region

Next, structural brain networks were constructed, as shown in Fig. 1. Automated Anatomical Labeling atlas was used to parcellate the brain into 90 regions [42], shown in Fig. 2. These regions correspond to nodes in the brain network. A link in the network corresponds to white matter tracts between two nodes. An unweighted network has a binary nature: a link between two nodes is either present or not. If there is at least one tract, the link is present. In addition to the binary network, a weighted network was also used in this study. In weighted networks, each link carries a numerical value representing a property of the connection between the two nodes. In this study, density-weight was used. It is calculated by dividing the total number of fibers between the two nodes by the mean volume of the two nodes. The same network weight was also used in the only previous network study investigating adults with ASD [8].

### Graph theoretical analysis

The graph theoretical measures used in this study are presented in Table 1. For more detailed information, please see Bullmore and Sporns [12] and Rubinov and Sporns [13]. The Brain Connectivity Toolbox was used to calculate the measures [13]. Both global properties (network-level) and local properties (node-level) were calculated for all individuals and for both the binary and the weighted networks. The global measures used were degree (or strength for the weighted networks), normalized clustering coefficient [43–45], normalized characteristic path length [43], and global efficiency [46]. Normalized clustering coefficient and normalized characteristic path length were calculated by comparing the networks and random networks with preserved weight, degree, and strength distribution [47]. Local measures used in this study were degree (or strength), clustering coefficient [43–45], local efficiency [46, 48], and betweenness centrality [49–51]. The network properties of the patients and the controls were compared with a two-sample *t* test. The node-level results were corrected for multiple comparisons with the number of the nodes, 90, by using a Bonferroni correction. Thus, the corrected *p* < 0.05 significance threshold is *p* < 0.00056. Possible differences in the laterality of the networks were also tested by constructing a separate network for the left and the right hemisphere for all subjects and both binary and weighted networks. The laterality index for the global measures was then calculated as follows: laterality index = (left − right)/(left + right). In addition, the biggest hubs were investigated for both patients and controls based on strength and betweenness centrality. The average strength or betweenness centrality was calculated for each node for each group, and then nodes that were larger than average by more than two standard deviations were selected as hubs.

## Results

We found global differences both in the binary and in the density-weighted networks between subjects with ASD and controls, as shown in Tables 2 and 3, respectively. In both networks, global efficiency was significantly decreased in subjects with ASD, and in the binary network, the normalized characteristic path length was increased in subjects with ASD. Strength was significantly decreased in subjects with ASD in the density-weighted networks.

**Table 2:** Results for the global properties of the binary network

Measures	Patients (mean ± standard deviation)	Controls (mean ± standard deviation)	*p* value
Degree	19 ± 1.8	20 ± 2.2	0.12
Normalized clustering coefficient	1.5 ± 0.079	1.5 ± 0.083	0.98
Normalized characteristic path length	1.1 ± 0.014	1.0 ± 0.0083	0.042
Global efficiency	0.58 ± 0.020	0.59 ± 0.017	0.042

**Table 3:** Results for the global properties of the density-weighted network

Measures	Patients (mean ± standard deviation)	Controls (mean ± standard deviation)	*p* value
Strength	6.6e−05 ± 5.1e−06	7.3e−05 ± 6.3e−06	0.0024
Normalized clustering coefficient	1.6 ± 0.088	1.6 ± 0.10	0.90
Normalized characteristic path length	1.1 ± 3.4e−02	1.1 ± 4.5e−02	0.22
Global efficiency	2.3e−06 ± 1.7e−07	2.5e−06 ± 1.6e−07	0.014

**Fig. 3:**
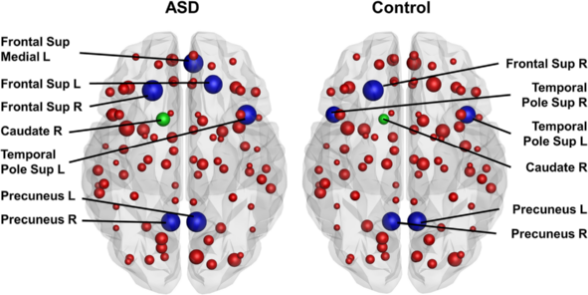
Local results in the binary networks. In the binary network, subjects with ASD had significantly higher betweenness centrality in the right caudate (*green node*) than the control subjects. The size of the nodes reflects the betweenness centrality of the node. The hubs are marked in *blue. Abbreviations*: *L* = left, *R* = right, *Frontal Sup Medial* = superior frontal gyrus, medial part, *Frontal Sup* = superior frontal gyrus, dorsolateral part, *Temporal Pole Sup* = temporal pole, superior temporal gyrus

**Fig. 4:**
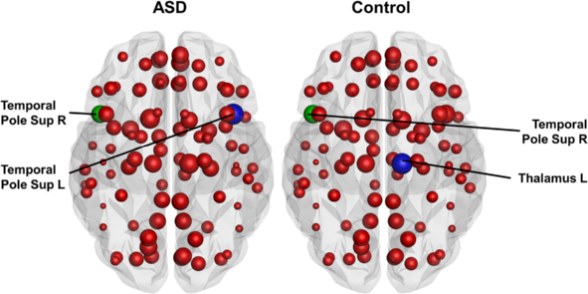
Local results in the weighted networks. In the density-weighted network, the strength of the right superior temporal pole (*green node*) was significantly lower in subjects with ASD than in control subjects. The size of the nodes reflects the strength of the node. The hubs are marked in *blue. Abbreviations*: *L* = left, *R* = right, *Temporal Pole Sup* = temporal pole, superior temporal gyrus

We also found local differences in both networks, as shown in Figs. 3 and 4. In the binary network, the betweenness centrality of the right caudate was significantly higher (*p* = 0.000094) in subjects with ASD (167 ± 51) than in the control subjects (96 ± 41). In the density-weighted network, the strength of the right superior temporal pole was significantly lower (*p* = 0.00041) in subjects with ASD (25 ± 4.5) than in control subjects (30 ± 5.4). These results survived a Bonferroni correction for multiple comparisons. Other *p* values are reported in Additional file 1: Table S1.

The following hubs were identified in the binary networks of both groups: the right superior frontal gyrus, dorsolateral part (Frontal Sup R); the left and right precuneus (Precuneus L, Precuneus R); the left temporal pole; and the superior temporal gyrus (Temporal Pole Sup L), as shown in Fig. 3. Moreover, patients had two additional hubs, the left superior frontal gyrus, dorsolateral part (Frontal Sup L), and the left superior frontal gyrus, medial part (Frontal Sup Medial L), and controls had one additional hub: the right temporal pole, superior temporal gyrus (Temporal Pole Sup R). In the weighted networks, only one hub for each group was identified, as shown in Fig. 4. The patients’ hub was the left temporal pole, superior temporal gyrus (Temporal Pole Sup L), whereas the controls’ hub was the left thalamus (Thalamus L). In Figs. 3 and 4, the size of the nodes reflects the betweenness centrality or strength of the node, respectively. The hubs are marked in blue and the regions of significant local differences between the groups in green.

In addition, the laterality of both networks was investigated at a global level. There was a significant difference (*p* = 0.037) in the laterality of the normalized characteristic path length between the groups in the binary network. The difference between the left and the right networks was increased in subjects with ASD (−0.0041 ± 0.0063) compared to controls (−0.00045 ± 0.0032). No global differences were found in the laterality in the density-weighted network.

## Discussion

Structural brain networks of adults with high-functioning ASD and age-, sex-, and IQ-matched healthy controls were compared both at the global and local levels by analyzing binary and density-weighted networks. In both networks, global efficiency was significantly lower in subjects with ASD. In addition, in the binary networks, the normalized characteristic path length was increased, and in the density-weighted networks, the strength was significantly decreased in subjects with ASD. In the node-level analyses of the binary networks, the betweenness centrality of the right caudate was significantly increased in subjects with ASD, and in the density-weighted networks, the strength of the right superior temporal pole was significantly decreased in subjects with ASD. Although there are some differences in the results found in binary and weighted networks, it is not surprising as the network structure is highly different in them. In the binary networks, all existing connections have the same weight of one, while in the weighted networks some of them may have a weight close to zero, and others have very high weights. Nevertheless, with both approaches the global results indicate decreased integration of the structural brain networks in ASD.

It has been hypothesized that the neurobiological deficits in ASD are related to abnormalities in the connectivity of the brain [1]. More specifically, recent studies, comprehensively reviewed in [52], suggest that the long-distance connectivity is reduced and the short-distance and local connectivity is increased in ASD [52]. Our finding of reduced global efficiency in subjects with ASD, both in the binary and the density-weighted networks, supports these theories. In addition, we also compared characteristic path length between the two groups. While these two variables are inversely related, global efficiency is mainly affected by shorter paths while characteristic path length by longer paths [13]. Characteristic path length was longer in the subjects with ASD in the binary network, suggesting abnormal integration of the structural brain networks in subjects with ASD but not in the density-weighted network. Thus, this could indicate that in the weighted networks, the shorter paths contribute more to the abnormal connectivity in ASD than the longer paths. Moreover, the overall strength was significantly lower in the density-weighted networks in subjects with ASD compared to controls, supporting the theory of global underconnectivity. However, we did not find evidence of local overconnectivity in subjects with ASD, as no differences were found in the clustering coefficient either in the binary or the density-weighted networks.

In our previous studies based on the same subjects, we found increased fiber coherence, measured by fractional anisotropy, in subjects with ASD both globally [37] and locally [28], being most prominent in the left inferior longitudinal fasciculus. However, increased fiber coherence does not necessarily mean that the brain networks would be functionally more efficient. It has previously been suggested that because of difficulties in differentiating signal from noise, strong physical connectivity and low computational connectivity could actually reinforce each other [1]. This theory fits our results of increased fiber coherence and reduced global efficiency in subjects with ASD. Furthermore, a study performed in an overlapping sample showed that the brains of subjects with ASD were functionally more asynchronous than in control subjects and that the functional connectivity was decreased in ASD between the frontal pole and several other regions [53].

In addition to the global analyses, we also performed node-level comparisons between the subjects with ASD and controls. In the binary networks, the betweenness centrality of the right caudate was significantly higher in subjects with ASD. This means that the caudate nucleus is more central to information transfer in subjects with ASD than in controls. In addition, there were differences in the clustering coefficient and the local efficiency in the caudate nucleus, but these results did not endure the Bonferroni correction. The caudate belongs to the basal ganglia, which are connected to the cortex via cortico-striatal loops [54]. Together with the putamen, the caudate receives most of the input from the cortex to the basal ganglia [54]. The caudate nucleus has been related to repetitive and stereotyped behavior and to obsessive-compulsive disorder, Tourette syndrome, and autism [55]. In autism, volume changes of the caudate nucleus have been reported in many studies [55]. A recent study showed positive correlations with bilateral caudate nuclei volume for compulsive and ritual behaviors in autism [56]. In obsessive-compulsive disorder, caudate hyperactivity and hypermetabolism have been reported [57, 58]. Our finding of increased centrality of the right caudate in the structural networks may also lead to its functional hyperactivity, although further experiments need to be performed to confirm this.

In the node-level analyses of the density-weighted network, the strength of the right superior temporal pole was significantly lower in subjects with ASD. The right temporal pole is associated with emotions and socially relevant memories, whereas the left temporal pole is related to semantic memory [59]. Damage to the right temporal pole may induce emotional blunting, inability to be at ease in social company, and reduced ability to recognize famous or family faces [59]. Moreover, patients with temporal variation of frontal temporal dementia with right temporal pole atrophy may become introverted, cold, and lack empathy [59]. In addition, the temporal pole is thought to have an important role in the theory of mind network [59]. Many of these symptoms fit well with the clinical manifestation of Asperger syndrome. All of the node-level differences between the subjects with ASD and controls both in the binary and the density-weighted networks are reported in Additional file 1: Table S1, but the other results are not discussed here as they did not endure the strict Bonferroni correction for multiple comparisons.

There is only one previous study in which graph theoretical approach was used to analyze brain networks of adults with ASD [8]. Like in this study, the authors used density-weight as a weight for the links. Our results are in accordance with their results, as they found reduced nodal local efficiency in ASD in many areas. The subjects were male, like in this study, and the mean age was slightly higher (34.4 ± 10.67 years) than in our sample (28.6 ± 5.7 years). The main difference was that the IQ in their sample (verbal IQ 88.5 ± 23.1, performance IQ 106.1 ± 15.9) was significantly lower than in our sample (verbal IQ 125.1 ± 15.3, performance IQ 121.9 ± 12.9). Moreover, they used traditional DTI-based tractography while we used a novel CSD-based approach able to reliably distinguish crossing fiber configurations unlike DTI [23–25].

The laterality of the brain networks has been previously investigated in healthy subjects [60]. The left hemisphere was found to be more efficient than the right hemisphere, whereas betweenness centrality and small-worldness values were higher in the right hemisphere. We investigated possible differences in the lateralization between subjects with ASD and controls and found a significant difference in the normalized characteristic path length indicating a bigger difference in the integration of the left and right networks in patients than in controls [13]. This means that in autism, the normalized characteristic path length is longer in the right hemisphere than in the left hemisphere, which indicates that the left hemisphere is better integrated than the right and the difference is significantly larger than in controls. This suggests that in our sample of adults with high-functioning ASD, the left hemisphere, which is known to be more involved in language processing and logical reasoning, may be better able to combine information from distributed brain regions [13] compared to the right hemisphere, which is relatively more involved in intuitive processing.

A limitation of this study concerns the lack of Autism Diagnostic Interview—Revised and Autism Diagnostic Observation Schedule, which are standard instruments in the diagnostics of ASD in many countries. In Finland, they were not in standard use at the time of diagnosis, and therefore, we do not have this information for our ASD subjects. However, the questionnaires used in this study (autism spectrum quotient, empathy quotient, and systemizing quotient) have been specifically designed for individuals with high-functioning ASD, and both individuals with ASD and controls were thoroughly screened to exclude other psychiatric disorders. Another limitation is a relatively small sample size. However, Finland is an isolated and genetically homogeneous country [61], which can be beneficial as heritable factors affect white matter organization [62]. Furthermore, the DW MRI acquisition used in this study was suboptimal for CSD-based tractography [39, 63]. Nevertheless, using CSD is highly beneficial in comparison to DTI, as CSD is able to reliably identify fiber crossings, present in 60–90 % of white matter voxels [21]. For optimal acquisition parameters, we refer to [63]. As ASD is a heterogeneous and multifactorial disorder, our results cannot be extended to the whole spectrum. Therefore, further structural brain network studies should be performed to also investigate the lower end of the ASD spectrum and children and adolescents with ASD.

## Conclusions

We found global and local differences in the brain networks between subjects with ASD and age-, sex-, and IQ-matched healthy controls. These networks were found to be less efficient in ASD, and in the node-level analyses, there were differences in two regions, the right caudate and the right superior temporal pole, associated with impairments typical in ASD. Our study was the first network study in ASD in which CSD-based tractography was used. We strongly believe that high-angular resolution diffusion imaging acquisition and analysis techniques such as CSD should be used in all future studies, as the standard diffusion tensor model is insufficient in correctly identifying multiple distinct fiber orientations in a voxel.

### Ethics, consent, and permissions

The ethics committee of Hospital District of Helsinki and Uusimaa approved of the research protocol, and all participants signed a written informed consent form before participating in the study.

## Additional file

Additional file 1: Table S1.
*p* values for the local network properties. *p* values <0.05 are emphasized.

## Abbreviations

ASD: autism spectrum disorder
CSD: constrained spherical deconvolution
DTI: diffusion tensor imaging
DW: diffusion-weighted
MRI: magnetic resonance imaging
