# Supplementary material for: Abnormal wiring of the connectome in adults with high-functioning autism spectrum disorder
Source: Mol Autism. 2015 Dec 15;6:65. doi: 10.1186/s13229-015-0058-4 (PMC4681075; doi:10.1186/s13229-015-0058-4)
Supplement: Additional file 1: Table S1. — p values for the local network properties. p values <0.05 are emphasized. [file 13229_2015_58_MOESM1_ESM.pdf]

Supplementary table 1. P-values for the local network properties. P-values < 0.05 are emphasized.

Weight	Network measure	Precentral L	Precentral R	Frontal Sup L	Frontal Sup R	Frontal Sup Orb L	Frontal Sup Orb R	Frontal Mid L	Frontal Mid R	Frontal Mid Orb L	Frontal Mid Orb R
Density-weight	Betweenness centrality	**0.01783**	0.54073	0.65398	0.60049	0.62918	0.38087	0.84056	0.94463	0.10758	0.46629
	Strength	0.05219	0.12718	0.34802	0.08038	0.43833	**0.00408**	0.32784	0.10641	0.20036	0.07695
	Clustering coefficient	0.58070	0.21563	0.25770	0.53923	0.17856	0.71723	0.10221	0.42446	**0.01537**	0.75086
	Local efficiency	0.96718	0.23154	0.70773	0.23785	0.72089	0.08398	0.71723	0.34060	**0.00611**	0.31309
Binary	Betweenness centrality	0.08376	0.93094	**0.03860**	0.87128	0.35843	0.06068	0.37495	0.42170	0.24290	0.92099
	Degree	0.05779	0.37963	0.35112	0.56280	0.15349	**0.03733**	0.23629	0.39677	0.32177	0.10785
	Clustering coefficient	0.37076	0.58331	0.23649	0.68118	0.44464	0.35487	0.17786	0.80251	**0.01401**	0.55546
	Local efficiency	0.16856	0.58392	0.23830	0.50942	0.95948	0.99231	0.18740	0.81235	**0.00650**	0.22560

Weight	Network measure	Frontal Inf Oper L	Frontal Inf Oper R	Frontal Inf Tri L	Frontal Inf Tri R	Frontal Inf Orb L	Frontal Inf Orb R	Rolandic Oper L	Rolandic Oper R	Supp Motor Area L	Supp Motor Area R
Density-weight	Betweenness centrality	NaN	0.14764	0.80545	0.90914	0.14647	0.80662	NaN	0.25023	0.14956	0.42089
	Strength	0.29257	0.30396	**0.01807**	0.09528	0.54571	0.97476	0.05343	0.08849	0.34827	0.17487
	Clustering coefficient	0.50282	0.46699	0.32751	0.19077	0.06310	0.47246	0.50519	0.90552	0.70269	0.30132
	Local efficiency	0.68058	0.57071	0.31049	**0.01755**	0.34890	0.91486	0.26136	0.32042	0.65281	0.27472
Binary	Betweenness centrality	0.35256	0.27811	0.77035	0.29744	0.67431	0.15694	0.40553	0.82166	0.56307	0.25025
	Degree	0.39252	0.40590	0.05865	0.34382	0.76098	0.72268	0.08811	0.15762	0.42388	0.57622
	Clustering coefficient	0.33530	0.78338	0.65145	0.63319	0.15054	0.33830	0.92337	0.69582	0.93326	0.76251
	Local efficiency	0.62317	0.76265	0.36408	0.23987	0.31119	0.22277	0.86121	0.86223	0.89743	0.34934

Weight	Network measure	Olfactory L	Olfactory R	Frontal Sup Medial L	Frontal Sup Medial R	Frontal Med Orb L	Frontal Med Orb R	Rectus L	Rectus R	Insula L	Insula R
Density-weight	Betweenness centrality	0.82968	0.39923	0.84712	0.06620	0.93076	0.20668	0.35689	0.22287	0.35078	0.71139
	Strength	0.27458	0.63124	0.30433	0.94715	0.45917	0.07087	0.06405	0.10052	0.43348	0.37611
	Clustering coefficient	0.21266	0.37376	**0.02538**	0.19313	0.42613	0.79621	0.66980	0.88900	**0.00924**	0.07815
	Local efficiency	**0.04583**	0.70108	0.28232	0.30660	**0.00988**	0.39126	0.14015	**0.02677**	**0.01148**	0.10263
Binary	Betweenness centrality	0.59912	0.91097	**0.02741**	0.42636	0.72697	0.24348	0.13780	0.13078	0.11699	0.35949
	Degree	0.53497	0.69188	0.12155	0.52535	0.91929	0.29154	0.23809	0.23129	0.60679	0.86714
	Clustering coefficient	0.75274	0.18523	**0.04317**	0.35961	0.75880	0.24251	0.95401	0.89369	0.07027	0.23819
	Local efficiency	0.36299	0.37706	**0.04062**	0.47714	0.65933	0.68405	0.55721	0.25280	0.06542	0.18569

Weight	Network measure	Cingulum Ant L	Cingulum Ant R	Cingulum Mid L	Cingulum Mid R	Cingulum Post L	Cingulum Post R	Hippo-campus L	Hippo-campus R	ParaHippo-campal L	ParaHippo-campal R
Density-weight	Betweenness centrality	0.73945	0.98191	0.12604	0.10398	NaN	0.25023	0.52124	0.66197	0.05519	0.42031
	Strength	0.75045	0.56237	**0.00391**	0.14999	0.88835	0.83369	0.06515	0.63844	0.72433	0.69138
	Clustering coefficient	0.05904	0.11597	0.27242	0.86454	0.41490	0.92400	0.36230	0.45261	0.92169	0.40617
	Local efficiency	0.21498	0.20267	0.60032	0.64887	0.21798	0.07812	0.83796	0.92838	0.79800	0.39552
Binary	Betweenness centrality	0.36516	0.11621	**0.01761**	0.68703	0.64385	0.51818	0.17175	0.90969	0.64822	0.67184
	Degree	0.45052	0.32434	**0.02651**	0.15960	0.38677	0.67012	0.15471	0.71793	0.54273	0.93173
	Clustering coefficient	0.11394	0.30841	**0.03010**	0.41064	0.50394	0.25541	0.44430	0.52574	0.94602	0.66001
	Local efficiency	0.12828	0.32513	**0.03086**	0.51264	0.35018	0.60108	0.50209	0.71559	0.99937	0.67395

Weight	Network measure	Amygdala L	Amygdala R	Calcarine L	Calcarine R	Cuneus L	Cuneus R	Lingual L	Lingual R	Occipital Sup L	Occipital Sup R
Density-weight	Betweenness centrality	0.25774	0.48968	0.21027	0.77907	0.75317	0.10623	0.82464	0.67203	0.89734	0.60467
	Strength	0.43562	0.28584	0.80567	0.58412	0.54338	0.66116	**0.01558**	0.10920	0.95877	0.37333
	Clustering coefficient	0.21545	0.66608	**0.01142**	0.42057	0.36445	0.74387	0.82326	0.53227	0.79276	0.16205
	Local efficiency	0.12369	0.59626	0.05018	0.33353	0.25836	0.36803	0.33324	0.21666	0.86031	0.47519
Binary	Betweenness centrality	0.31623	0.46072	**0.03208**	0.41854	0.21260	0.68587	0.12825	0.74513	0.35651	0.47904
	Degree	0.37148	0.30238	0.24829	0.94769	0.51793	0.90704	0.23352	0.81410	0.53208	0.20363
	Clustering coefficient	0.75820	0.81584	0.32522	0.95244	0.93907	0.27219	0.06319	0.91922	0.36904	0.38652
	Local efficiency	0.57325	0.34438	0.31059	0.82334	0.80384	0.22225	0.05753	0.89486	0.29440	0.50652

Weight	Network measure	Occipital Mid L	Occipital Mid R	Occipital Inf L	Occipital Inf R	Fusiform L	Fusiform R	Postcentral L	Postcentral R	Parietal Sup L	Parietal Sup R
Density-weight	Betweenness centrality	0.67738	0.24596	0.98721	0.24655	0.27476	0.18854	0.48180	0.27335	0.33375	0.12503
	Strength	**0.03904**	0.53828	0.14669	0.08061	0.43424	**0.03139**	**0.01338**	**0.04532**	0.72839	0.10433
	Clustering coefficient	0.32011	**0.02067**	0.21072	0.13042	0.87746	0.82856	0.79096	0.79242	0.07700	0.85685
	Local efficiency	0.13240	0.31856	0.50134	**0.04583**	0.96762	0.51287	0.41903	0.76812	0.26856	0.95218
Binary	Betweenness centrality	0.78924	0.20722	0.08279	0.95878	0.32060	0.16807	0.12276	0.20239	**0.01639**	0.14734
	Degree	0.37888	0.26761	0.21137	0.53476	0.30010	**0.03570**	0.10567	0.08016	0.73296	0.10525
	Clustering coefficient	0.71663	**0.03569**	0.27690	0.16844	0.59276	0.76179	0.38506	0.93103	0.11538	0.60377
	Local efficiency	0.63287	0.09536	0.23113	0.22985	0.66117	0.99919	0.23922	0.91204	0.05568	0.57891

Weight	Network measure	Parietal Inf L	Parietal Inf R	Supra-Marginal L	Supra-Marginal R	Angular L	Angular R	Precuneus L	Precuneus R	Paracentral Lobule L	Paracentral Lobule R
Density-weight	Betweenness centrality	0.40896	0.12044	0.31715	0.24726	NaN	0.11022	0.84519	0.19267	0.08804	0.28482
	Strength	0.09525	0.13676	0.47906	0.09177	0.54924	0.15084	0.19046	0.10287	0.84623	0.93195
	Clustering coefficient	0.90719	0.44006	0.59290	0.11610	0.99884	0.08400	0.29707	0.29425	0.61545	0.14917
	Local efficiency	0.42155	0.34572	0.15863	0.13159	0.40405	0.09868	0.23925	0.14266	0.68092	0.66136
Binary	Betweenness centrality	0.45880	0.33620	0.74049	0.14171	0.41280	0.93318	0.49464	0.56419	0.45118	0.21015
	Degree	0.23604	0.20236	0.56672	0.29221	0.82257	0.13674	0.47061	0.72533	0.53020	0.96570
	Clustering coefficient	0.17216	0.56270	0.37716	0.62619	0.42992	0.11642	0.85111	0.70631	0.31511	0.06682
	Local efficiency	0.12287	0.44664	0.22008	0.80050	0.57462	0.10558	0.48225	0.76804	0.18162	0.05356

Weight	Network measure	Caudate L	Caudate R	Putamen L	Putamen R	Pallidum L	Pallidum R	Thalamus L	Thalamus R	Heschl L	Heschl R
Density-weight	Betweenness centrality	0.56032	**0.02491**	0.43607	0.18028	0.63630	0.23280	0.62111	0.11095	NaN	NaN
	Strength	**0.02091**	0.72034	**0.03148**	**0.02659**	0.48931	0.72776	**0.00617**	0.05150	**0.00235**	0.92480
	Clustering coefficient	0.74506	**0.00074**	0.57960	**0.01145**	0.14610	0.53894	0.51453	0.81502	0.15900	0.86000
	Local efficiency	0.80029	**0.01210**	0.17262	0.06570	0.58495	0.73695	0.74135	0.83023	**0.02147**	0.91943
Binary	Betweenness centrality	0.22623	**0.00009**	0.16593	0.93675	0.99044	0.22927	0.28918	0.41768	0.97567	0.41203
	Degree	**0.01825**	0.05140	0.25273	0.12753	0.73779	0.64610	**0.04043**	**0.04526**	**0.02631**	0.87178
	Clustering coefficient	0.46129	**0.03615**	0.08812	0.13558	0.08292	0.77905	0.22239	0.76097	0.29798	0.91294
	Local efficiency	0.65312	**0.02598**	0.05178	0.15231	0.08108	0.85696	0.28628	0.87218	0.11046	0.91124

Weight	Network measure	Temporal Sup L	Temporal Sup R	Temporal Pole Sup L	Temporal Pole Sup R	Temporal Mid L	Temporal Mid R	Temporal Pole Mid L	Temporal Pole Mid R	Temporal Inf L	Temporal Inf R
Density-weight	Betweenness centrality	0.11877	**0.01420**	0.57316	0.70725	0.21558	0.52037	0.48473	0.85574	0.16261	0.30222
	Strength	**0.04803**	0.31967	0.84773	**0.00040**	**0.03630**	0.19478	0.08136	0.34303	0.17607	**0.01740**
	Clustering coefficient	0.99807	0.05823	0.09745	0.75164	0.44300	0.44360	0.28154	0.56965	0.67291	0.78134
	Local efficiency	0.46189	0.06369	0.09883	0.45258	0.09113	0.37996	0.20435	0.92757	0.50574	0.75526
Binary	Betweenness centrality	0.56327	**0.04113**	0.18084	**0.04744**	0.96604	0.55565	0.41570	0.45331	0.92754	0.41820
	Degree	0.05405	0.78278	0.29386	**0.00946**	0.51305	0.19555	0.07415	0.21025	0.22392	**0.03561**
	Clustering coefficient	0.67420	0.18786	0.53636	0.27549	0.72546	0.99382	0.61598	0.71798	0.87647	0.49075
	Local efficiency	0.97497	0.21397	0.96156	0.37015	0.59343	0.97682	0.36679	0.68421	0.91870	0.69580
